# A Monte Carlo and experimental investigation of the dosimetric behavior of low‐ and medium‐perturbation diodes used for entrance *in vivo* dosimetry in megavoltage photon beams

**DOI:** 10.1120/jacmp.v13i6.3917

**Published:** 2012-11-08

**Authors:** Mohammad Amin Mosleh‐Shirazi, Sareh Karbasi, Daryoush Shahbazi‐Gahrouei, Shahram Monadi

**Affiliations:** ^1^ Physics Unit Department of Radiotherapy and Oncology and Centre for Research in Medical Physics and Biomedical Engineering Shiraz University of Medical Sciences Shiraz Iran; ^2^ Department of Medical Physics Isfahan University of Medical Sciences Isfahan Iran

**Keywords:** *in vivo* dosimetry, diode correction factors, low‐perturbation diodes, treatment verification, Monte Carlo simulation

## Abstract

Full buildup diodes can cause significant dose perturbation if they are used on most or all of radiotherapy fractions. Given the importance of frequent *in vivo* measurements in complex treatments, using thin buildup (low‐perturbation) diodes instead is gathering interest. However, such diodes are strictly unsuitable for high‐energy photons; therefore, their use requires evaluation and careful measurement of correction factors (CFs). There is little published data on such factors for low‐perturbation diodes, and none on diode characterization for 9 MV X‐rays. We report on MCNP4c Monte Carlo models of low‐perturbation (EDD5) and medium‐perturbation (EDP10) diodes, and a comparison of source‐to‐surface distance, field size, temperature, and orientation CFs for cobalt‐60 and 9 MV beams. Most of the simulation results were within 4% of the measurements. The results suggest against the use of the EDD5 in axial angles beyond ±50° and exceeding the range 0° to +50° tilt angle at 9 MV. Outside these ranges, although the EDD5 can be used for accurate *in vivo* dosimetry at 9 MV, its CF variations were found to be 1.5–7.1 times larger than the EDP10 and, therefore, should be applied carefully. Finally, the MCNP diode models are sufficiently reliable tools for independent verification of potentially inaccurate measurements.

PACS numbers: 87.10.Rt; 87.50.cm; 87.55.km; 87.56.Fc

## I. INTRODUCTION

Several factors contribute to the accuracy of the delivered dose to patients in external‐beam radiotherapy (EBRT). Although some of these factors can be checked by implementing suitable quality control procedures, *in vivo* dosimetry is a highly recommended method for verification of the accuracy of dose delivery in EBRT.^1^, ^2^ *In vivo* dosimetry is particularly valuable because any discrepancies between the expected results and those found by *in vivo* dosimetry can highlight an overall error (resulting from one or more sources of error in the preceding processes) in the dose delivered.^3^, ^4^ By finding any significant differences between prescribed and delivered doses, the probability of errors from different sources (e.g., patient setup, machine output, selection of energy or wedge) can be surveyed to assess the problem, and attempts made to remove it.^5^, ^9^ In addition, to make sure that critical organs do not receive doses exceeding tolerance levels, *in vivo* dosimetry can be applied to measure the dose received by those critical organs inside or outside of the treatment field.^10^, ^14^

Besides diodes, thermoluminescent dosimeters (TLDs) and metal‐oxide semiconductor field‐effect transistors (MOSFETs) can also be used for *in vivo* dosimetry.^(15)^ Although TLDs, for instance, can yield quite accurate and precise results in well‐controlled conditions ^(16)^, the characteristics of diodes, such as their high sensitivity, excellent reproducibility, good mechanical stability, small size, and online reading, distinguishes them for *in vivo* dosimetry.^(17)^ Diode response depends on instantaneous dose rate, field size, temperature, energy, and orientation.^18^, ^22^ As a result, in addition to measuring the calibration coefficient, measurements of relevant correction factors (CFs) are necessary to account for the differences in diode response in calibration and clinical conditions. According to the American Association of Physicists in Medicine (AAPM) Task Group 62 Report, the correction factors, ${\text{CF}}_{x}$, are derived from the following formula:^(15)^

(1) $$CF_{X}=\frac{D_{w}(X)}{R(X)}/\frac{D_{w}(cal)}{R(cal)}$$

where $D_{w}(\text{cal})$ and $D_{w}(X)$ are the doses to water at the depth of maximum dose, $d_{\text{max}}$, under reference and X conditions, respectively, and *R(cal)* and *R(X)* are the diode readings under their respective stated conditions. Temperature and orientation dependencies are intrinsic, and variation of these factors does not involve a change in beam characteristics. Thus these correction factors are derived from the following simpler equation:^(15)^

(2) $$CF_{tem,angle}=\frac{R(cal)}{R(X)}$$

Another factor of concern is dose perturbation produced by a diode when it is used in the treatment field. The dose perturbation caused by a full buildup diode can be high, even as much as 13%.^(23)^ Therefore, a significant perturbation of the target dose may occur if it is used on most or all of the treatment fractions. Given the importance of performing *in vivo* dosimetry in most or all of complex treatment fractions, using thin buildup (low‐perturbation) diodes instead is gathering interest.^(24)^ However, such diodes are strictly unsuitable for dosimetry of high‐energy megavoltage photons. Therefore, their use requires evaluation, and careful measurement and application of additional CFs that are larger than usual.^(24)^

There are several situations in which accurate diode *in vivo* dosimetry is impractical or impossible in routine clinical practice. In such situations, diode *in vivo* dosimetry is sometimes used by medical physicists, but with large uncertainties arising from the fact that measurement of the necessary correction factors is very difficult or impossible experimentally.

One of those situations is out‐of‐field measurements. Thin buildup diodes may be used in this situation but before diode dosimetry in out‐of‐field measurements, its calibration and/or measurement of correction factors against an ionization chamber is required. However, not knowing the correct chamber calibration factor for the spectrum outside the field poses a problem for this calibration. Medical physicists may ignore such out‐of‐field spectral changes in practice. The rationale for such a simplification would be that, as dose levels are relatively low outside the field, even large percentage errors in dosimeter response will be small in magnitude in absolute terms, and often may not lead to clinically relevant dose uncertainties. Diodes are sometimes used clinically in or near the beam penumbra region (e.g., lens dose assessment in lateral whole‐brain fields). Because of the steepness of the dose gradient within the penumbra, accurate determination of CFs for this situation is again difficult. Ideally, a solution enabling more accurate patient dose assessment using a method that does not suffer from measurement uncertainties is desirable. As a solution to the problems faced in diode dosimetry in situations like those mentioned above, designing a reliable Monte Carlo (MC) model of the implemented diode(s) will be helpful. Such an MC model may be used to determine a diode's CFs in such conditions, or serve as a tool to perform an independent check of their measurement. The model may also be useful when trying to explain the observed behavior of the diode in question.

Several investigators have characterized commercial diodes experimentally and measured their CFs at various energies,^3^, ^17^, ^25^, ^27^ but there is little published data on such factors for low‐perturbation diodes. Also, to the best of our knowledge, there is no published data on diode characterization for 9 MV photons.

The aims of the present study were: (i) comparison of experimentally measured CFs for source‐to‐skin distance (SSD), field size, temperature, and orientation (axial and tilt) for two commercial low‐ and medium‐perturbation diodes for use in entrance *in vivo* dosimetry in cobalt‐60 and 9 MV X‐rays; (ii) design and testing of MC models of two commercial diodes in 9 MV X‐rays; and (iii) to use the model to provide some information to help explain the observed trends in diode response with changes in field size.

## II. MATERIALS AND METHODS

### A. Materials and experimental setups

**Figure 1:**
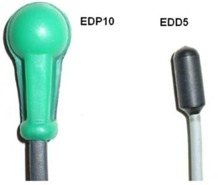
The shapes of the diodes used in this study (courtesy of IBA Dosimetry).

Two types of commercial diode dosimeters (IBA Dosimetry GmbH, Schuarzenbruck, Germany) and a DPD‐3 electrometer by the same manufacturer were used in this study. The diodes used were two hemispherical‐shape EDP10 devices with epoxy and stainless steel buildup and an EDD5 cylindrical‐shape dosimeter with epoxy and polystyrene buildup (Fig. 1). The water equivalent thicknesses of each of the two diode's buildups were 10 mm and 4.5 mm, respectively, as stated by the manufacturer. Except for the measurement of sensitivity variation with temperature (SVWT), we stacked and abutted $30\times 30\times 1\;{\text{cm}}^{3}$ solid water slabs to make a $60\times 60\times 15\;{\text{cm}}^{3}$ phantom large enough for investigation of field size, SSD, and orientation correction factors.

**Figure 2:**
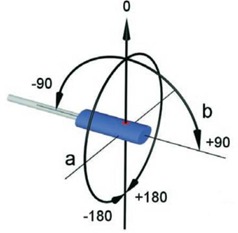
Directional dependence (a=axial; b=tilt).

Measurements were made in the 9 MV X‐rays of a Neptun 10pc linear accelerator (ZDAJ, Warsaw, Poland) and a Phoenix cobalt‐60 unit (Best Theratronics, Kanata, Canada). We used a Scanditronix FC65‐G ion chamber (IBA Dosimetry GmbH, Schuarzenbruck, Germany) and a Wellhofer Dose 1 electrometer by the same manufacturer to measure the dose to water at $d_{\text{max}}$, when required. The chamber had been calibrated based on absorbed dose to water by a secondary standards dosimetry laboratory implementing the International Atomic Energy Agency TRS‐398 protocol. Acceptance tests and measurements of calibration and correction factors were carried out based on the recommendations of the AAPM.^(15)^

To ensure uniform irradiation to all diodes while reducing the effects of scattered radiation from one diode to the others in the measurements of field size and SSD CFs, all diodes were positioned on the phantom surface at least 1 cm apart, centered about the beam's central axis. The ion chamber was placed at the depth of $d_{\text{max}}$ of the beam (2.2 cm for 9 MV and 0.5 cm for cobalt‐60). The field size was changed from $5\times 5{\;\text{cm}}^{2}$ to the maximum $40\times 40{\;\text{cm}}^{2}$ for 9 MV and to $35\times 35{\;\text{cm}}^{2}$ for cobalt‐60, while the standard SSD was set at 100 cm and 80 cm, respectively. The SSD was changed from 80 cm to 120 cm for 9 MV and from 63 cm to 100 cm for cobalt‐60, while a standard $10\times 10{\;\text{cm}}^{2}$ field size was set.

According to Eq. (2), ion chamber measurements were not required for investigation of orientation CFs and SVWT. To measure the axial (diode long axis perpendicular to the gantry rotation) and tilt (diode long axis parallel to the gantry rotation) angle CFs (Fig. 2),^(28)^ the gantry angle was changed in each case first from 0° to 80° and then from 0° to ‐80°.

**Figure 3:**
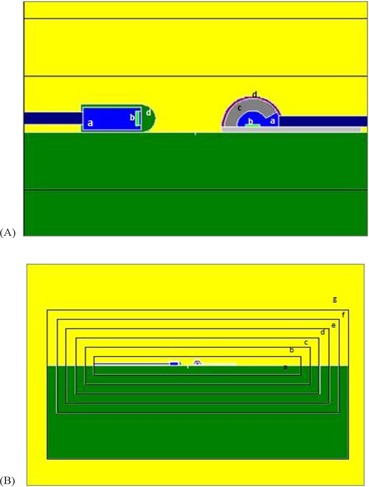
Schematic diagrams of the simulated diodes (A) (a=epoxy; b=silicon die; c=stainless steel buildup; d=cover); and the importance (imp) regions defined around them (B) (importance values for regions a, b, c, d, e, f, and g being 1536, 768, 384, 192, 96, 48, and 16, respectively).

To measure SVWT, we used a $25\times 25\times 30\;{\text{cm}}^{3}$ water tank. The diodes were taped on a thin sheet of Perspex which, in turn, was in contact with the water in the phantom. The temperature was increased from about 20°C to 40°C using heaters, while a homogenizer ensured a uniform water temperature within the volume. A calibrated digital thermometer with a flat sensor was affixed on the Perspex sheet to monitor the surface temperature. The standard SSD and field size were set in each case.

Fig. 2. Directional dependence (a=axial; b=tilt).

### B. Monte Carlo simulations

MC simulation was performed using the MCNP code version 4C^(29)^ with the help of information on the geometry and materials of each diode as supplied by their manufacturer, following the signing of a nondisclosure agreement. Our previously simulated and validated model of the linear accelerator with 9 MV X‐rays was used as our source.^(30)^ About 2 billion particles were transported with a cut off energy of 10 keV for photons and 521 keV for electrons. The phantom and diode arrangements were simulated exactly as the experimental setup. Due to the smallness of the silicon die's volume, in order to improve precision, we used the geometry splitting with Russian roulette function for variance reduction. Accordingly, additional imaginary cells were defined around the diodes to obtain the ability of gradual increase of the silicon die's importance^(29)^ (Fig. 3). A surface source was written at the end of the gantry head for each field size to obviate the need for repeated running of the histories regarding the same structures. We used this source for subsequent runs. By making appropriate changes in the geometry of the linac and coordinate transformation cards, different field sizes, angles, and SSDs were achieved. Using tally ^*^F8:p,e which calculates the energy deposition for photons and electrons, the dose absorbed in the whole of the silicon die of each diode was computed directly by dividing it by the mass of silicon die.

**Figure 4:**
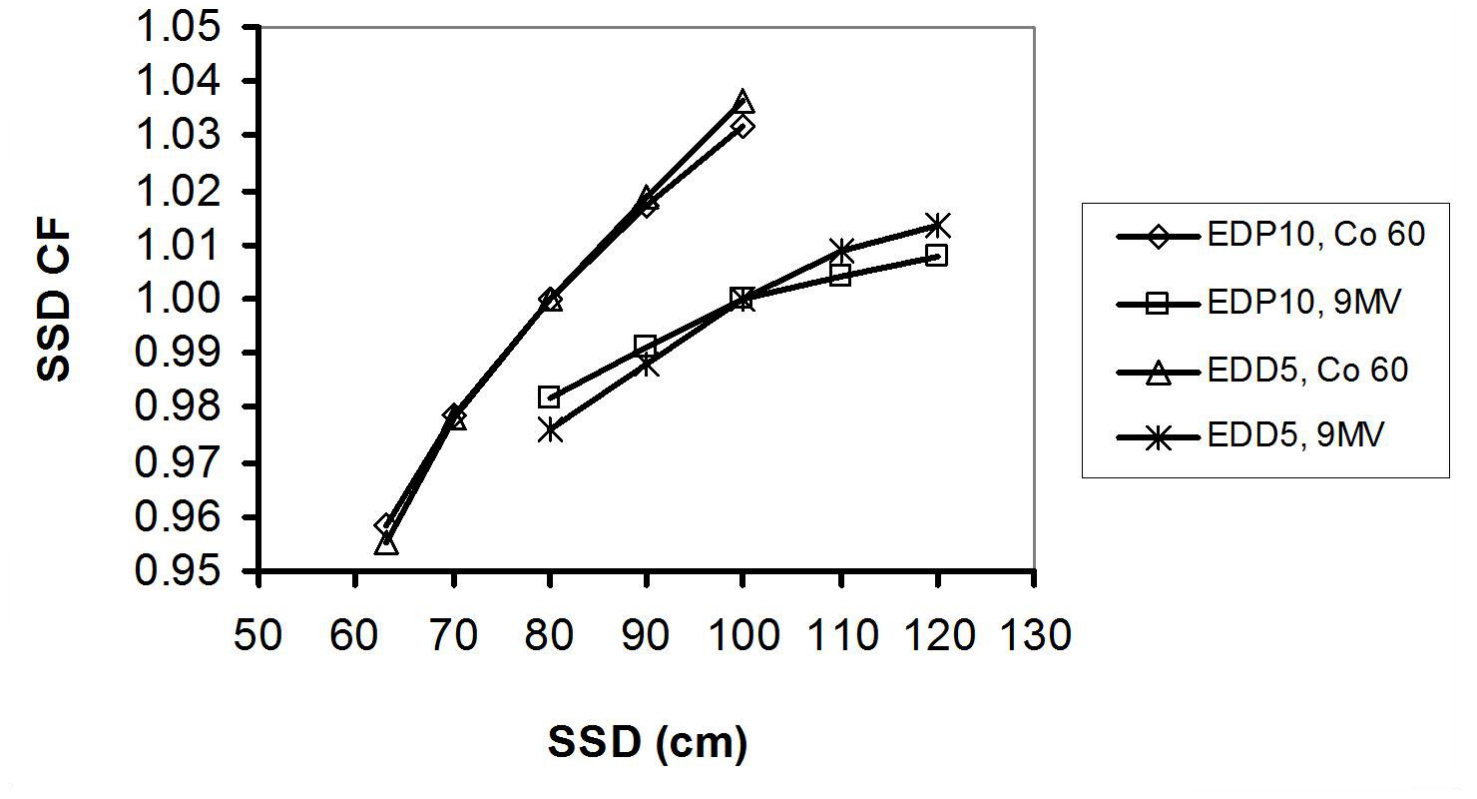
The SSD correction factors for EDP10 and EDD5 in cobalt‐60 and 9 MV X‐rays.

To compare the different scattered radiations seen by the chamber at $d_{\text{max}}$ and the diodes on the surface of the phantom in order to help explain the trends observed by the two types of dosimeters' responses with changes in field size, a separate program code was run. First, by defining a detector tally beyond the target, a photon source point (a photon source placed at the focal spot point, containing phase‐space information of the photons emanating from the target) was obtained by running about 2 billion linac electron histories. Then, in this code, the photon source point was run with two ring detectors (F5 tally), one placed at the $d_{\text{max}}$ and the other on the phantom surface. The energy flux of photons and electrons reaching these ring detectors were computed. As the printout for F5 tally is normally in two parts — (i) the total of all contributions, and (ii) the direct (or uncollided) contribution to the detector from the source — the scatter fractions (scatter‐to‐primary ratios) were derived from the tallies. Electrons were not tallied for this part of the study as F5 only tallies photons.

## III. RESULTS

As was previously mentioned, the aim of the comparisons between the MC simulations with measurements was to validate the MC models of the diodes. The aim did not include obtaining CFs for the diodes from MC simulations (instead of using direct ionization chamber measurements), as the recommended way to obtain CFs is through experimental ionization chamber measurements. Therefore, the results of the CF measurements and MC simulations have to be presented separately, as the former includes ionization chamber dose‐to‐water measurement $D_{W}(X)$ (Eq. (1)), but the latter does not. In the MC simulations relative values, normalized to a reference value, were compared. Therefore, numerically different results were obtained in the two cases which could not be compared directly.

### A. Experimental results

**Figure 5:**
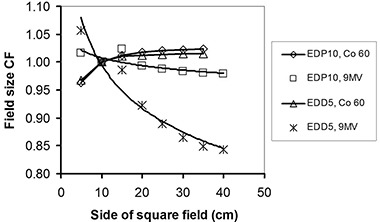
The field size correction factors for EDP10 and EDD5 in cobalt‐60 and 9 MV X‐rays.

**Figure 6:**
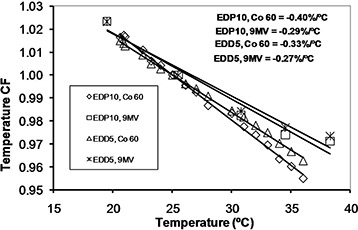
The temperature correction factors for EDP10 and EDD5 in cobalt‐60 and 9 MV X‐rays with linear fits to the data points.

**Figure 7:**
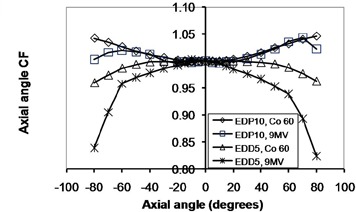
The axial angle correction factors for EDP10 and EDD5 in cobalt‐60 and 9 MV X‐rays.

**Figure 8:**
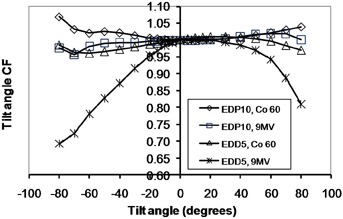
The tilt angle correction factors for EDP10 and EDD5 in cobalt‐60 and 9 MV X‐rays.

The SSD, field size, temperature, and axial and tilt obliquity CFs normalized to a reference value (SSD=100 cm for 9 MV and SSD=80 cm for cobalt‐60, field size $=10\times 10{\;\text{cm}}^{2}$, tilt and axial angles=0°, and temperature=25°C) are plotted in Figs. 4 to 8.

### B. Monte Carlo simulations

**Figure 9:**
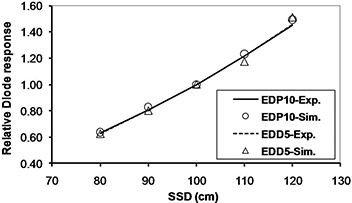
Monte Carlo calculation of diode response at different SSDs in 9 MV X‐rays.

**Figure 10:**
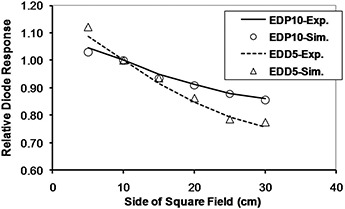
Monte Carlo calculation of diode response for different field sizes in 9 MV X‐rays.

**Figure 11:**
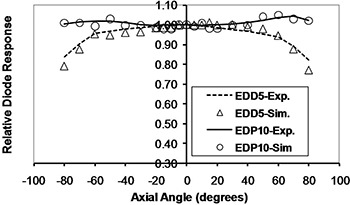
Monte Carlo calculation of diode response at different axial angles in 9 MV X‐rays.

**Figure 12:**
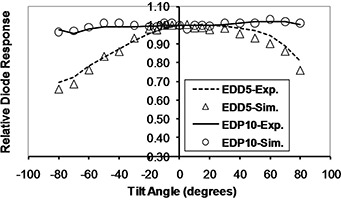
Monte Carlo calculation of diode response at different tilt angles in 9 MV X‐rays.

The simulation results for diode response dependence on SSD, field size, and obliquity, normalized to a reference value (SSD=100 cm, field size $=10\times 10{\;\text{cm}}^{2}$, tilt and axial angles=0°), are plotted in Figs. 9 to 12. In these plots “Exp.” and “Sim.” stand for experimental and simulation results, respectively. The experimental measurements have an approximately 1% uncertainty, while uncertainty in the simulations reached up to 5% in some cases, due to limited computer resources.

The results of the simulations to compare the scattered radiation reaching the chamber at $d_{\text{max}}$ and the diodes on the phantom surface revealed that the scatter fraction at the surface was 8.6%, while it was 12.0% at $d_{\text{max}}$.

## IV. DISCUSSION

The current report presents the results of measuring and comparing SSD, field size, temperature, and orientation correction factors for a low‐ (EDD5) and a medium‐perturbation (EDP10) diode in cobalt‐60 and 9 MV beams, as well as designed Monte Carlo models of these diodes. There is no published data on characterization of these diodes for 9 MV X‐rays, but there are some published results for 6, 10 and 18 MV X‐rays (summarized in Table 1, together with the results of this study). At 9 MV and within the ranges of the parameters studied here, compared to the EDP10, the EDD5 CF variations were found to be 1.5, 5.1, 4.0 and 7.1 times larger for SSD, field size, axial angle, and tilt angle, respectively.

**Table 1:** EDP10 and EDD5 characterization results of this work (9 MV and cobalt‐60) and previously published data for 6, 10, and 18 MV X‐rays (SSD=80 cm to 120 cm, $\text{field}\;\text{size}=5\times 5\;{\text{cm}}^{2}$ to $40\times 40\;{\text{cm}}^{2}$, $\text{angle}=‐80^{{}^{\circ}}$ to 80°).

*Diode Type*	*SSD CF Variation*	*Field Size CF Variation*	*Angular CF Variation*	*SVWT/°C*
EDP10	$6\;\text{MV}:1{\%}^{\left(27\right)}$	$6\;\text{MV}:1.5{\%}^{\left(28\right)}$	$6\;\text{MV}:\text{Axial}:2.5{\%}^{\left(28\right)}$ $\text{Tilt}:3{\%}^{\left(28\right)}$	$\text{Co}‐60:0.36{\%}^{\left(26\right)}$
	$18\;\text{MV}:2{\%}^{\left(27\right)}$	9 MV: 4.2%^a^	9 MV: Axial: 4.3%^a^, Tilt: 4.3%^a^	$6\;\text{MV}:0.38{\%}^{\left(26\right)}$
	9 MV: 2.6%^a^	Co‐60^c^:5.9%^a^	Co‐60: Axial: 4.6%^a^, Tilt: 6.9%^a^	$0.26‐0.34{\%}^{\left(28\right)}$
	Co‐60^b^: 7.3%^a^			9 MV: 0.29%^a^
				Co‐60: 0.40%^a^
EDD5	$6\;\text{MV}:6{\%}^{\left(24\right)}$	$6\;\text{MV}:20{\%}^{\left(24\right)}$	$6\;\text{MV}:17.5{\%}^{\left(24\right)}$	9 MV: 0.27%^a^
	$10\text{MV}:11{\%}^{\left(24\right)}$	$10\text{MV}:36{\%}^{\left(24\right)}$	$10\;\text{MV}:23{\%}^{\left(24\right)}$	Co‐60: 0.33%^a^
	9 MV: 3.7%^a^	9 MV: 21.5%^a^	9 MV: Axial: 17.6%^a^, Tilt: 30.7%^a^	
	Co‐60^b^: 8.1%^a^	Co‐60^c^: 4.7%^a^	Co‐60: Axial: 4%^a^, Tilt: 3.9%^a^	

^a^ The results of this study

^b^ SSD=63 cm‐100 cm

^c^ $\text{field}\;\text{size}=5\times 5\;{\text{cm}}^{2}$ ‐ 35 times 35 cm^2^.

### A. SSd dependence of diode response

The variations of SSD CFs for cobalt‐60 were 7.3% and 8.2% and for 9 MV were 2.6% and 3.8%, for EDP10 and EDD5, respectively. SSD dependence is due to the variation of instantaneous dose rate, electronic contamination and the thickness of the diode's buildup.^(15)^ A higher response occurs at higher dose rates and higher electronic contamination of shorter SSDs. Further, a thicker buildup can reduce electronic contamination and, consequently, decrease the diode response. In this study, both diodes had a decreasing sensitivity with increasing SSD at both energies, but the variation of EDD5 CFs in 9 MV X‐rays was larger than EDP10 due to the thickness of buildup in EDD5 being insufficient for electronic equilibrium at 9 MV The simulation results for SSD dependence were in good agreement with measurements, with maximum differences of 2.9% and 3.5% for EDP10 and EDD5, respectively.

### B. Field size dependencies

The variations of field size CF for cobalt‐60 energy were 6.0% and 4.7% and for 9 MV were 4.2% and 21.4%, for EDP10 and EDD5, respectively. The difference in electronic contamination and scatter radiation received by a diode on the surface and an ionization chamber at $d_{\text{max}}$ causes a variation in CFs with field size.^(31)^ Both ascending and descending variation of CFs with field size are reported for different diodes at various energies.^3^, ^28^, ^32^ In this study, the field size CF for both diodes had ascending variation for cobalt‐60 and descending variation at 9 MV. With reference to Eq. (1), this correction factor is dependent of the ratio $D_{W}$(X)/R(X), where the dose to water at field size *X* ($D_{W}$ *(X))* is proportional to chamber reading, and *R(X)* is the diode reading. In cobalt‐60 radiation for which the buildup thicknesses in both diodes are sufficient for electronic equilibrium, the diodes which are placed at the surface do not see as much scatter as the chamber at $d_{\text{max}}$ sees and, therefore, CF is greater than 1. At 9 MV, neither diode has sufficiently thick buildup, and both see a larger amount of head scatter than usual; this decreases the difference in the scatter fraction seen by diodes and the chamber at different depths. Therefore, the CF of the EDP10 diode with a thicker buildup is around unity. The buildup of the EDD5 diode is far too thin for 9 MV X‐rays and, therefore, its CF is much lower than unity. The higher diode‐to‐chamber relative response in the thinner buildup diode (EDD5), in comparison with EDP10, may be attributed to higher detection probability of very low‐energy, multiple‐scattered photons and electron contamination that are more abundant in larger fields.

The simulations also confirmed this behavior of diodes at 9 M V, where their results had maximum differences of 1.6% and 3.2% with measurements for EDP10 and EDD5, respectively. The large variation of this factor as a function of field size for EDD5 at 9 MV is due to the insufficient buildup for electronic equilibrium.

### C. temperature dependencies

The SVWT values in cobalt‐60 energy were 0.40%/°C and 0.33%/°C and in 9 MV were 0.29%/°C and 0.27%/°C, for EDP10 and EDD5, respectively. The sensitivity variation of diode response with temperature is due to the lifetime of carriers and their mobility.^(15)^ The lifetime of carriers increases by increasing temperature,^(15)^ but their mobility decreases.^18^, ^33^ The sensitivity of the diodes in this study, like those in most reports, increased with temperature. It means that the effect of growing lifetime with temperature was greater than the decreased carrier mobility. The SVWT was larger in cobalt‐60 than 9 MV for both diodes, which is in agreement with the reported results of different temperature dependence of EDP diodes at various energies.^(26)^

### D. Orientation dependencies

The percentage variations of obliquity CFs were as follows (EDP10, EDD5): axial angle in cobalt‐60 energy (4.6%, 4.0%); axial angle in 9 MV X‐rays (4.4%, 17.6%); tilt angle in cobalt‐60 energy for negative angles (6.9%, 3.9%); tilt angle in cobalt‐60 energy for positive angles (4.0%, 3.0%); tilt angle in 9 MV energy for negative angles (4.3%, 30.7%); tilt angle in 9 MV energy for positive angles (2.0% and 19.1%).

As expected, both diodes had good symmetry in positive and negative directions. The axial angular CFs for EDP10 at both energies and directions increased with increasing angle, while this CF decreased for EDD5 in both energies and directions. Due to the alignment of the silicon die (vertical in EDD5 and horizontal in EDP10, as shown in Fig. 3) as well as the different shapes of these diodes, the observed difference in behavior is logical. The variation of this CF at 9 MV was larger for EDD5 than EDP10 due to the insufficient buildup of the former for electronic equilibrium. The tilt angular CF at positive angles (center to head of diode) was different from the negative angles (center to tail of diode), because the beam passes through different structures in the diode when approaching from different directions. The variation of tilt angle CF for EDD5 at 9 MV was larger than EDP10, again due to insufficient buildup.

The ascending and descending trends for EDP10 and EDD5 CFs were in close agreement to those predicted by MC simulation. The maximum difference between the simulation and measurement results for axial angle dependence was 2.3% for EDP10 at all angles and 3.3% for EDD5 up to angles ±70°. The maximum difference for tilt angle was 2% for EDP10 at all angles and 4.9% for EDD5 up to angles ±70°. The obliquity dependence of diode response is due to backscatter from phantom, patient, or diode tail and buildup thickness.^(15)^ The results show that phantom backscattering, low‐energy photons, and electron contamination had more effect on the EDD5 with a thin buildup as the gantry angle increased. It must be noted, however, that differences in phantom attenuation and scatter occur as the gantry rotates, which combines with the diodes' own anisotropic response to produce the measured obliquity factors.

## V. CONCLUSIONS

The CFs for EDD5 are large in 9 MV X‐rays, which arises from the insufficient buildup thickness to achieve electronic equilibrium. Within the ranges of the parameters studied, its CF variations were found to be 1.5 to 7.1 times larger than the EDP10. For example, the field size CF variation was found to be as high as 21.5% when using EDD5 at 9 MV. Accordingly, care should be taken when using the EDD5 at energies around 9 MV or higher. The results of the present study are in general agreement with a previous report which concluded that the EDD5 may be applied as a low‐perturbation diode at high energies (particularly for the lower megavoltage beam energies) and for angles of obliquity below 45°.^(24)^ Our results suggest against the use of this diode in axial angles beyond ±50° and exceeding the range 0° to +50° tilt angle at 9 MV, because a small error in setup for such angles may cause large errors in the diode readings. The angular tilt CF variations can be as large as 30.7% with EDD5 at 9 MV. Finally, having obtained reasonably good agreement between the simulations and measurements, the model can be used as a sufficiently reliable tool for independent verification of potentially inaccurate measurements.

## ACKNOWLEDGEMENTS

The authors would like to thank IBA Dosimetry GmbH for providing the detailed information on the EDD5 and EDP10 diodes required for the simulations. This work was financially supported by a grant from Shiraz University of Medical Sciences, Shiraz, Iran (grant No. 90‐01‐01‐3210).
